# Setting Dengue Fever Epidemic Thresholds Between 2016 and 2021 in the Central Health Region, Burkina Faso: An Ecological Study

**DOI:** 10.1007/s44197-023-00137-w

**Published:** 2023-07-11

**Authors:** Jean Claude Romaric Pingdwindé Ouédraogo, Sylvain Ilboudo, Espérance Ouédraogo, Wendlasida Thomas Ouédraogo, Salfo Ouédraogo, Benoit Césaire Samadoulougou, Mikaila Kaboré, Léon G. Blaise Savadogo

**Affiliations:** 1 Laboratoire de Développement de Médicament, Université Joseph Ki-Zerbo, Ouagadougou, Burkina Faso; 2 Laboratoire de Recherche-Développement de Phytomédicaments et Médicaments (LR-D/PM), Institut de Recherche en Sciences de la Santé (IRSS), Ouagadougou, Burkina Faso; 3 International Research Laboratory, Environnement, Santé et Sociétés (IRL 3189, ESS), CNRST, Ouagadougou, Burkina Faso; 4 Direction Régionale de la Santé (DRS) du Centre, Ouagadougou, Burkina Faso; 5 Country Coordinating Mechanism of Global Fund to Fight Against AIDS, Tuberculosis and Malaria, Ouagadougou, Burkina Faso; 6 Centre Hospitalier Régional de Ziniaré, Ziniaré, Burkina Faso; 7 Institut Supérieur des Sciences de la Santé, Université NAZI BONI, Bobo-Dioulasso, Burkina Faso

**Keywords:** Dengue fever, Threshold, Mean, Median, Cumulative sum

## Abstract

**Background:**

Dengue fever (DF) is endemic in Burkina Faso, with 70% of its burden supported by the Central Health Region. Then, a single confirmed case can no longer mean an epidemic. This study aimed at describing trends and setting epidemic thresholds of DF in the Central Health Region.

**Data and Methods:**

An ecological study was conducted using monthly data from DF surveillance between 2016 and 2021. Three methods were applied to set alert and intervention thresholds of DF monthly incidence rate: mean [mean + 2 SD], median [3rd quartile] and cumulative sum (C-sum) [C-sum + 1.96 SD]. These thresholds were plotted with the monthly incidence rates for 2021.

**Results:**

In total, 54,429 cases were reported between 2016 and 2021. Dengue cases increased biannually. The median annual incidence rate did not vary significantly across years [Kruskal–Wallis: *χ*^2^(5) = 9.825; *p* = 0.0803]. Within a year, the monthly incidence rate fell under 48.91 cases per 100,000 inhabitants between January and September and peaked in October or November. With the mean and C-sum methods, the 2021 monthly incidence rate remained below the intervention thresholds (Mean + 2 SD and C-sum + 1.96 SD). With the median method, the incidence rate exceeded the alert and intervention thresholds in July–September 2021.

**Conclusions:**

If the DF incidence varied within a year due to the seasons, it was relatively stable between 2016 and 2021. The mean and C-sum methods based on the mean were subject to extreme values, giving high thresholds. The median method seemed better for capturing the abnormal increase in dengue incidence.

## Introduction

Dengue fever virus, carrying four antigenically different serotypes (DEN-1, DEN-2, DEN3, and DEN-4), belongs to the *Flavivirus* family [1]. Dengue fever is widespread in semi-urban and urban areas from tropical and sub-tropical settings [2]. It is a significant public health issue that threatens about 50% of people with an epidemic potential worldwide. Globally, about 100–400 million people are infected every year [2]. Dengue fever can be severe due to hemorrhagic complications and liver and heart damage. It can be fatal if management is delayed and inadequate.

In Africa, the first epidemic of dengue fever occurred in 1779–1780, simultaneously with North America and Asia [3]. However, there is a lack of diagnostic tools and a poor surveillance system of dengue in Africa, leading to an underestimation of its burden, a poorly known epidemiology, and a misdiagnosis of malaria [3–5]. The first epidemic in Burkina Faso was reported in 1925 [4]. Studies done in 1985 and later confirmed that dengue fever is frequent and continuous in the country, according to the United States’ level of epidemic risk [5–8]. However, most studies have focused on either seroepidemiology of dengue fever or individual reports on ongoing epidemics [9–11]. Recently, a study assessed the space–time dynamics of dengue fever in Burkina Faso for a short period (2016–2019) [12]. Still, the epidemiology of dengue fever in Burkina Faso is not well-described and needs more attention [7]. Moreover, there is serological evidence of zika virus among blood donors in Ouagadougou and Bobo-Dioulasso—Burkina Faso—another Flaviviridae transmitted by *Aedes* vector [13].

According to the 2010 Technical Guidelines for Integrated Disease Surveillance and Response (ISDR) in the African Region, one suspected dengue fever represents an alert threshold, while a single confirmed case is an action or intervention threshold [14]. A suspected case calls for several actions, including investigating 5–10 suspected cases using acute or convalescent-phase blood samples [14]. However, considering the age of these guidelines, this approach can no longer be applied to endemic areas for dengue fever like Burkina Faso. Higher thresholds should then be defined to improve dengue fever control for such settings. According to the guidelines for IDSR in Burkina Faso, alert and intervention thresholds are necessary in the surveillance of epidemic diseases, but no threshold has been established for dengue in the country [15].

Thresholds are already well-established for malaria, another vector-borne disease. Three different approaches (mean, median and cumulative sum) have been validated for malaria [14, 16–18], and could help in understanding dengue epidemics. In effect, thresholds indicate when to intervene and facilitate monitoring programmes by managers [15].

In Burkina Faso, the Central Health Region alone concentrates at least 70% of the dengue burden from the 13 health regions in the country [19]. Therefore, this region is more suitable for setting the epidemic thresholds. In addition, the reports on dengue fever in Burkina Faso have significantly improved since 2016, forming the basis for long-term studies.

This study aimed to describe dengue fever trends and set epidemic thresholds between 2016 and 2021 in Burkina Faso.

## Data and Methods

### Study Setting

Situated in the heart of West Africa, Burkina Faso is a landlocked country, whose health system is structured into 13 health regions. Based on the population size, the Central Health Region is the biggest and includes the municipality of Ouagadougou (Capital city) and six rural municipalities (Saaba, Pabré, Komsilga, Komki-Ipala, Koubri et Tanghin-Dassouri) [20]. It comprises five health districts: Baskuy, Nongr-Massom, Sig-Nonghin, Bogodogo and Boulmiougou. The districts are structured into smaller entities: the Health and Social Promotion Centres (CSPS) and the Medical Centres (CM) at the primary level. Then, those entities refer the complicated patients to the Medical Centres with Surgical Antenna (CMA) [19]. In addition to these public health facilities, the region holds multiple private facilities. About 70% of the total cases of dengue fever in the country are reported in this region [19].

The Central Health Region covers an area of 2857 square kilometres, with 3,032,668 inhabitants reported in 2019 [21]. For the same period, the population age structure showed 30.97% males, 31.38% females and 31.38% children under 15 years [20].

### Study Design and Period

It was an ecological study using monthly secondary data from the dengue surveillance system in the five districts of the Central Health Region between 2016 and 2021.

### Data Source and Processing

Dengue data for 2016–2021 were retrieved from the online Health Data Depository of Burkina Faso (ENDOS-BF). Data included suspected or probable cases. Suspected cases were those diagnosed clinically by the presence of fever or history of a fever combined with symptoms, such as headaches, retro-orbital pain, myalgias, arthralgia, nausea, vomiting, abdominal pain, rash, bleeding manifestations and shock syndrome. In addition to clinical figures, cases with a positive rapid diagnostic test were classified as probable (Positive Antigen NS1 and/or Immunoglobulin M and/or Immunoglobulin G).

The data on dengue were validated by the National System of Health Information (SNIS) following a known process. The reports are sent to the five districts by the 5th of the month following the month for which the report was submitted. The data are entered into ENDOS-BF by the District Health Information and Epidemiological Surveillance Centres (CISSE) [19]. Then, the districts have until the 20th of the month following the month of the report to check the quality of the ENDOS-BF data. Following the districts, the Regional Health Directorate has until the 25th of the month following the month covered by the report to validate the data from the various districts in the region [19]. Finally, the Directorate of Sectoral Statistics at the Ministry of Health has 15 days to validate the data in ENDOS-BF before it is officially available in that database.

### Statistical Analyses

#### Basic Statistics

Central tendency and dispersion statistics were used to summarise dengue crude data: mean ± standard deviation (SD), median (IQR: Q1–Q3), minimum–maximum, and sum.

#### Incidence Rate of Dengue

Dengue fever incidence per month was calculated by dividing the targeted month’s cases by the population at mid-year for the concerned year. In the Central Health region, the following folks at mid-year were considered: 2,637,303 people, 2,744,666 people, 2,854,356 people, 2,966,307 people, 3,080,375 people and 3,218,294 people in 2016, 2017, 2018, 2019, 2020 and 2021, respectively. Then, the annual average incidence rate was calculated by summing the monthly incidence rate for the year and dividing the sum by 12.

All incidence rates were multiplied by 100,000 to get the monthly or annual incidence rate per 100,000 inhabitants.

#### Dengue Year-to-Year Variability

The normality of dengue incidence rate distribution was checked through the Shapiro–Wilk test at a significance level of 5%. We hypothesised in the null hypothesis that the incidence rate distribution was normal. The distribution was not normally distributed (*N* = 72; *W* = 0.65002; *V* = 22.041; *z* = 6.737; *p* < 0.00001).

Given that the normality assumption was violated, a Kruskal–Wallis’s analysis was applied to test if the median incidence rate varies between years over the study period at the significance level of 5%. Thus, it was hypothesised in the null hypothesis that the median incidence rate was equal across years; then, in the alternative one, at least one average annual incidence rate differs from the others.

#### Mean and Mean + 2 Standard Deviation Method

This approach was developed by Cullen et al. for malaria weekly and monthly data [16]. It is similarly applied to set dengue epidemic thresholds using weekly data with at least a 5-year baseline [22]. This study used dengue monthly incidence rates from 2016 to 2020 as a baseline (5 years). The mean and mean + 2SD figures of the incidence rates were calculated for every month and plotted along with the dengue figures for 2021. The mean and the mean + 2SD represented the lower and upper thresholds, and the area between them showed the normal incidence rate channel. When the 2021 incidence was above the upper limit, an unusual increase was triggered, and an outbreak was suspected.

The mean represented the alert threshold, and the mean + 2SD was the intervention threshold.

#### Median and Third Quartile Method

Median and third quartile incidence rates were calculated with the monthly incidence rate from 2016 to 2020 and plotted with the monthly incidence rate of 2021. They represented the lower and upper limits. An epidemic situation is alerted when the incidence rate exceeds the third quartile. When the incidence rate crosses the median, an epidemic is alerted; the 3rd quartile represents the intervention threshold.

#### Cumulative Sum at the 3-Months Moving Average Method

The C-sum was developed by the Centers for Disease Control and Prevention (CDC) and suggested by the WHO to set epidemic thresholds for malaria, accounting for its seasonality [17]. Based on that, we calculated the incidence rate cumulative sum (C-sum) with a 5-year baseline (2016–2020) for dengue fever. For the actual month, the C-sum was obtained using the dengue incidence rate from 2016 to 2020 of the previous and following months. For example, February C-sum was calculated by summing the incidence rate of January, February, and March from 2016 to 2020 and dividing by 15. Then, C-sum and refined C-sum + 1.96 SD were plotted with a monthly incidence rate of 2021. The simple C-sum represents the alert threshold with the C-sum method, while the C-sum + 1.96 SD was the intervention threshold.

Like the mean + 2SD, the 3rd quartile and the C-sum represented the expected maximum incidence rate [23] and are the intervention thresholds meant to set off adequate responses.

## Results

### Time Series of Dengue Cases Over the Study Period

**Fig. 1:**
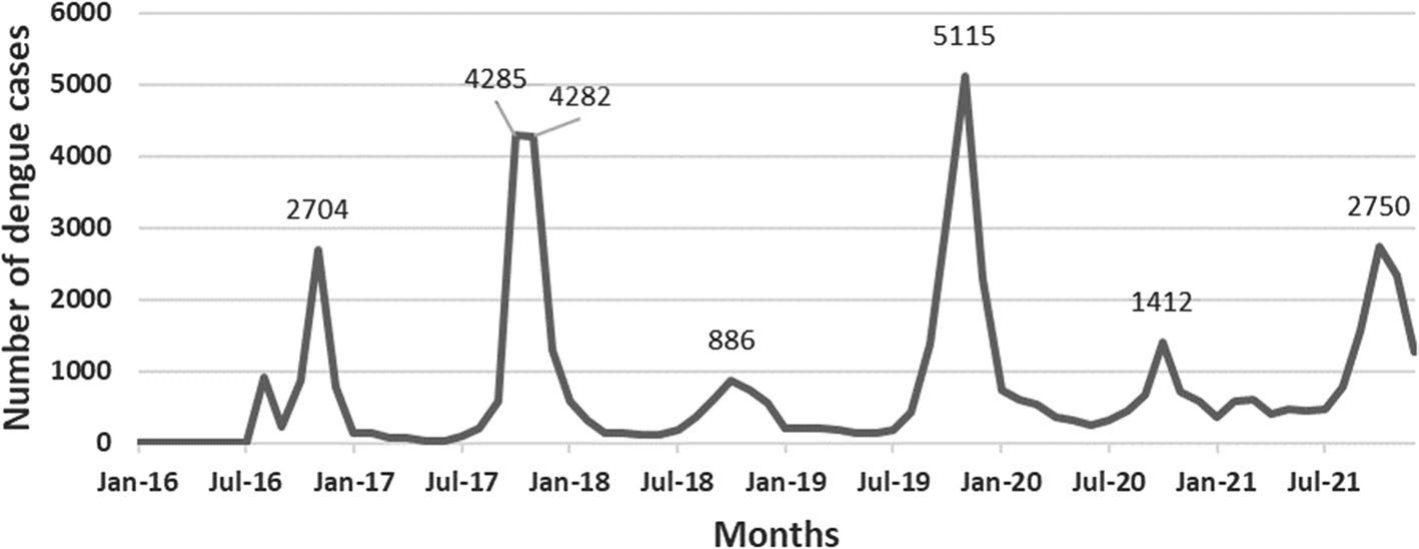
Evolution of dengue monthly cases between 2016 and 2021

Figure 1 shows the monthly distribution of dengue cases between January 2016 and December 2021. Dengue cases were reported in every month during the period. A 2-year periodicity has been spotted with maximum number of cases in October and November 2017 (4285 and 4282 cases), November 2019 (5115 cases) and October 2021 (2750 cases).

### Summary Statistics on Dengue Crude Data

Between 2016 and 2021, 54,429 dengue cases were reported in the Central Health Region, with monthly extremes of 3 cases (April and May 2016) and 5115 cases (November 2019). The total number of dengue cases varies biannually, exceeding 10,000 annually in 2017, 2019 and 2021. The mean number of cases also follows a biannual trend, with the highest mean number of cases in 2017 (939.08 cases), 2019 (1142.83 cases) and 2021 (1007.83 cases).

The median dengue cases varied from 11.5 (IQR: 4.5–829.5) in 2016 to 597.5 (IQR: 464–1426) in 2021. The median changed between 2016 and 2019, remaining under 350 cases for that period and exceeding 500 cases in 2020–2021. These basic statistics of dengue crude data are presented in Table 1.

**Table 1:** Central tendency and dispersion statistics on dengue fever between 2016 and 2021

Years	Mean ± SD	Median (IQR: Q1–Q3)	Minimum–maximum	Sum	Annual average incidence rate
2016	463.67 ± 799.30	11.5 (4.5–829.5)	3–2704	5564	17.58
2017	939.08 ± 1602.23	144.5 (73.5–940)	32–4285	11,269	34.21
2018	398.17 ± 271.69	342.5 (138.5–599.5)	113–886	4778	13.95
2019	1142.83 ± 1607.74	212 (182.5–1838)	132–5115	13,714	38.53
2020	584.17 ± 309.23	566 (346–696)	253–1412	7010	18.96
2021	1007.83 ± 812.40	597.5 (464–1426)	367–2750	12,094	31.30
Overall	755.96 ± 1052.82	419.5 (144.5–763.5)	3–5115	54,429	25.76

### Year-to-Year Variability of Dengue Incidence Rate From 2016 to 2021

According to the Kruskal–Wallis non-parametric test (results in Table 2), we were not confident enough [*χ*^2^(5) = 9.825; *p* = 0.0803] to conclude the equality of the different annual incidence rates across the 6 years. That is, there was no statistically significant difference in the median incidence rate between two or more of the 6 years.

**Table 2:** Results from the Kruskal–Wallis test comparing the annual incidence rate between 2016 and 2021

Years	Sample size	Median (Q1, Q3) incidence rate
2016	12	0.44 (0.17, 31.45)
2017	12	5.26 (2.68, 34.25)
2018	12	11.99 (4.85, 21.00)
2019	12	7.15 (6.15, 61.96)
2020	12	18.37 (11.23, 22.59)
2021	12	18.57 (14.42, 44.31)

*χ*^2^(5) = 9.825; *p* = 0.0803

### Intra-Year Variability of Dengue Incidence Rate Between 2016 and 2021

Dengue fever incidence is generally low from January to July and rises for the rest of the year. Up to September, the monthly incidence rate lies under 25 cases per 100,000 inhabitants most of the years, except in August 2016 (35.23 cases per 100,000 inhabitants), September 2019 (46.66 per 100,000 inhabitants) and September 2021 (48.91 cases per 100,000 inhabitants).

**Fig. 2:**
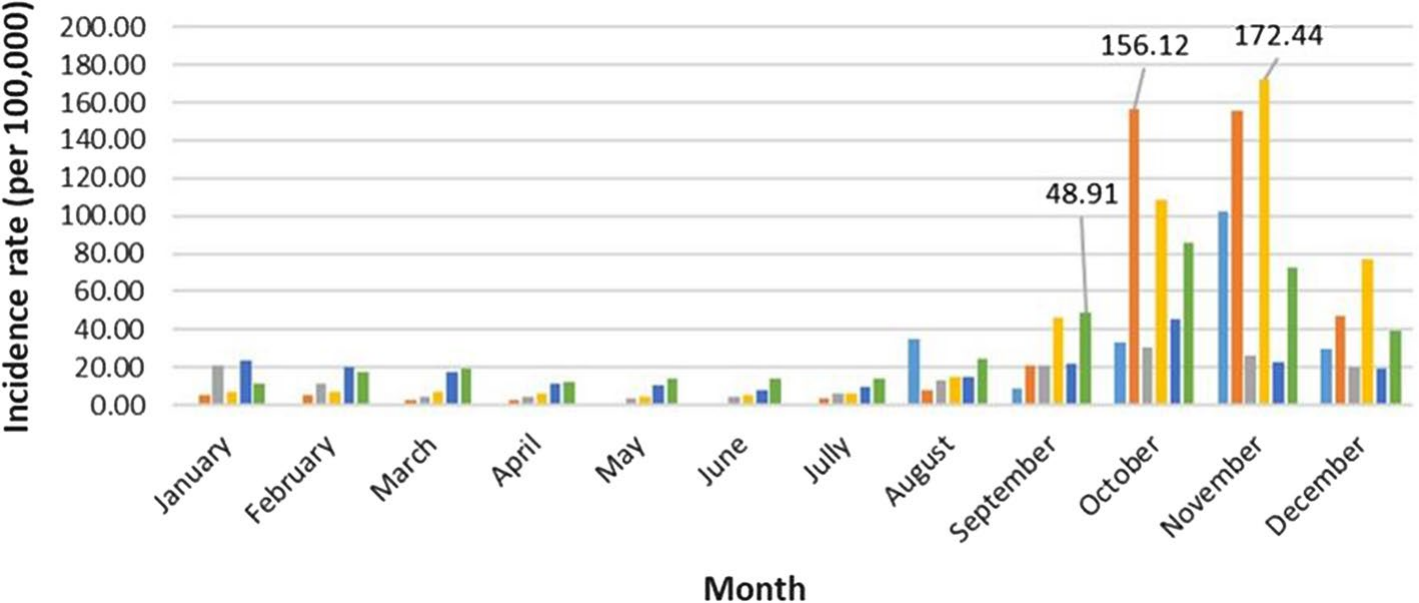
Dengue fever monthly epidemic curve between 2016 and 2021

The dengue incidence rate is high between October and December, peaking in October or November over the study period. The monthly incidence rate peaked in November 2016 and 2019, with 102.53 cases per 100,000 inhabitants and 172.44 cases per 100,000 inhabitants, respectively. For the years 2018 (31.04 cases per 100,000 inhabitants), 2020 (45.84 cases per 100,000 inhabitants) and 2021 (85.4 cases per 100,000 inhabitants), it peaked in October. With 156.12 cases per 100,000 inhabitants in October and 156.01 cases per 100,000 inhabitants in November, dengue showed almost a bimodal trend in 2017. The dengue fever monthly epidemic curve is shown in Fig. 2.

### Dengue Epidemic Threshold by the Mean and Median Methods

**Fig. 3:**
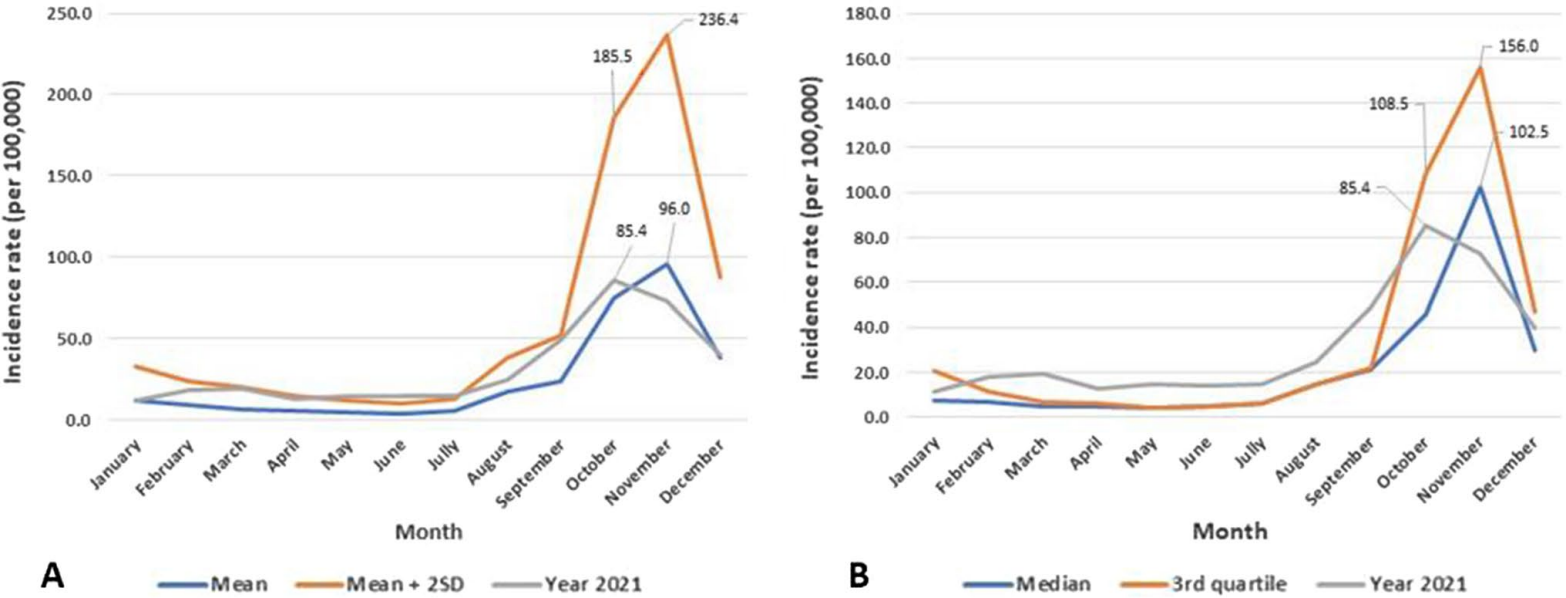
Graph of dengue fever mean (**A**) and median (**B**) thresholds with the 2021 incidence rate

Given that the yearly incidence rate did not vary across years, it was possible to use the 2016–2020 data as base data to set the dengue epidemic threshold in the Central Health Region. Epidemic thresholds based on the mean (A) and median (B) methods are plotted in Fig. 3.

Dengue monthly incidence rate of 2021 was confounded with the mean, mean + 2 SD, median and the 3rd quartile from January to July. Between July and September, the 2021 incidence rate fell between the mean and the mean + 2 SD and above the median and 3rd quartile.

From September to October, the 2021 incidence rate crosses the alert threshold, over the mean on one hand; over the median on the other hand. At the peak in November, the incidence rate for 2021 was below the mean and median thresholds. The thresholds in October were 185.5 per 100,000 inhabitants for the mean + 2 SD and 108.5 per 100,000 inhabitants for the 3rd quartile. All thresholds peaked in November at 236.4 per 100,000 inhabitants and 156.0 per 100,000 inhabitants, respectively, for the mean + 2 SD and the 3rd quartile. The incidence rate of 2021 fell below the alert and intervention thresholds (mean + 2 SD and 3rd quartile) between October and December.

### Dengue Epidemic Threshold by C-Sum

**Fig. 4:**
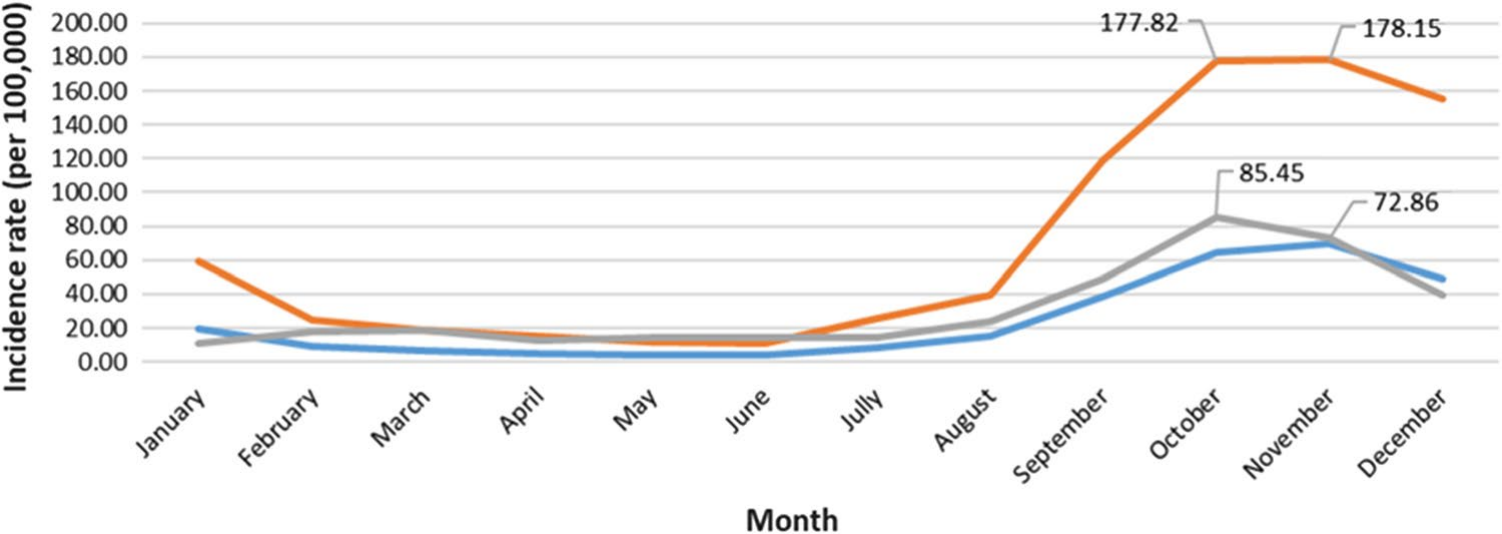
Graph of dengue fever C-sum thresholds with the 2021 incidence rate

Up to June, the 2021 incidence rate was almost confounded with the C-sum and C-sum + 1.96 SD thresholds. Then, it exceeded the C-sum without crossing the refined C-sum until November. The upper limits were 180.13 cases per 100,000 inhabitants in October and 180.36 cases per 100,000 inhabitants in November. Alert and intervention thresholds based on the C-sum method are shown in Fig. 4.

### Levels of the Different Thresholds Based on the Three Methods

All alert and intervention thresholds are presented in Table 3. The mean and mean + 2 SD thresholds are high between August and December, picking at 96.0 and 236.4 cases per 100,000 inhabitants in November. The most elevated thresholds occurred between September and December, culminating at 102.5 and 156.0 cases per 100,000 people in October, respectively, for the median and the third quartile. Respectively, the C-sum and C-sum + 1.96 were higher between September and December, highest in October at 69.8 and 178.1 cases per 100,000 people. In 2021, the highest monthly incidence rates were noted between August and December, picking in October with 85.4 cases per 100,000 people.

**Table 3:** Alert and intervention thresholds of incidence rates according to the three methods (incidence rate: cases per 100,000 people)

	Jan	Feb	Mar	Apr	May	Jun	Jul	Aug	Sept	Oct	Nov	Dec
2021	11.4	18.0	19.1	12.6	14.5	14.3	14.6	24.3	48.9	85.4	72.9	39.7
Mean	11.6	8.7	6.4	5.3	4.0	3.9	5.3	17.1	24.0	74.9	96.0	38.6
Mean + 2 SD	32.3	23.4	20.0	14.1	12.2	10.2	12.5	38.1	51.7	185.5	236.4	87.3
Median	7.3	7.0	4.8	4.9	4.0	4.5	6.0	14.6	21.5	45.8	102.5	29.7
3rd quartile	20.9	11.1	6.8	6.3	4.4	5.1	6.2	14.9	22.1	108.5	156.0	47.0
C-sum	19.6	8.9	6.8	5.2	4.4	4.4	8.8	15.5	38.7	65.0	69.8	48.7
C-sum + 1.96 SD	59.3	24.6	18.6	14.9	11.6	11.1	25.8	39.8	118.8	177.8	178.1	154.9

*Jan january,* Feb* february,* Mar* march, *Apr* april, *May* may, *Jun* june,* Jul* july,* Aug* august,* Sept* september,* Oct* october;* Nov* november;* Dec* december

## Discussion

This study aimed to describe dengue fever trends from 2016 to 2021 and set an epidemic threshold in the Central Health Region of Burkina Faso, using a 5-year baseline. In effect, the region bears about 70% of dengue burden of the country, probably due to its 2 million 4 hundred inhabitants, representing 12% of the country population and accounting for 45.4% of the urban population in 2019 [21]. We found a non-significant year-to-year variability and a seasonal pattern within the year. An intervention threshold was reached between July–September based on the median method, while the 2021 incidence rate never exceeded the intervention thresholds with the mean and the cumulative sum methods.

Following the dengue fever epidemic in 2013, the National Health System started reporting dengue fever cases in 2014 in the Central Health Region. The reporting system has improved since 2016, allowing for a step back in the data. Dengue fever is endemic in the Central Health Region, as cases have been reported every month over the study period. In addition, the annual incidence rate fell between 10 and 100 cases per 100,000 people most of the years during the study period, meaning a moderately endemic pattern [12]. Dengue fever incidence rose every 2 years, with an incidence rate exceeding 31 cases per 100,000 people in 2017, 2019 and 2021, about twice the incidence rate (13–19 cases per 100,000 inhabitants) of the other years. Similarly, dengue fever followed a 2-year cyclical epidemic pattern in Singapore, switching between DEN-1 and DEN-2 [24]. Studies have confirmed the circulation of serotypes DEN-1, DEN-2, DEN-3 and DEN-4 in Burkina Faso [10, 11]. Then, it was established that over 2014–2017, DENV-2 predominated during the outbreak period, while DENV-3 was most frequent before the outbreak, suggesting differences in dengue serotypes circulating over time [11]. However, dengue serotypes are not routinely collected, given that it is not cost-effective for cases management. Such data lacks from passive surveillance to allow further interpretation of dengue cyclical evolution. On one hand, epidemiological surveillance combined with sentinel surveillance could allow monitoring the types of dengue virus circulating one year after the other in Burkina Faso. On the other hand, the health system should be accordingly prepared to manage a higher number of cases in the Central Health Region, given this biannual trend.

Dengue incidence was relatively stable from January to July and increased for the rest of the year, almost concurrent with the arrival of the rainy season. Similarly, dengue serology was more frequently positive in the rainy season than during the dry season in Nigeria [25]. This seasonal trend is because dengue fever is a vector-borne disease primarily transmitted by *Aedes aegypti* mosquitoes and, to a lesser extent, *Ae. Albopictus* [2]. The availability of mosquito habitats is critical to the survival and development of larvae, and the reproduction of adult *Aedes* meant to transmit dengue fever [26, 27]. During the rainy season, rainfall and temperature ensure the habitats necessary for immature *Aedes* mosquitoes to survive and adults to breed [28, 29]. Ambient temperature is crucial to dengue fever transmission as it affects the development, survival, and reproductive behaviour of the *Aedes* vector and the replication of the dengue virus in the infected vector [29–31]. The favourable weather conditions contributed to dengue's peak in October or November. Similar trends were found in 2016 [32] and between 2016 and 2019 [12] in Ouagadougou at the end of the rainy season, when there was no heavy rainfall to drown the mosquito larvae. The seasonality of dengue incidence requires long-term time series to analyse the interactions of meteorological elements, such as temperature, precipitation, and humidity with dengue in Burkina Faso. A study on a short period (2017–2019) found maximum and minimum temperature, relative humidity, and wind speed to have a significant non-linear effect on dengue cases in the Central Health Region, explaining 83% of the case variance [33]. Non-linear effects of weather, based on mathetimatical models, were also shown using *Aedes* entomological parameters or patient-based data [34, 35].

As dengue is at least moderately endemic for every month between 2016 and 2021, one confirmed dengue case might no longer define an epidemic. In effect, a suspected dengue fever case was considered an alert threshold, while a confirmed one was an intervention threshold [14]. Simple methods such as mean, median and cumulative sum have been applied to set epidemic thresholds in malaria epidemic areas [14, 16, 17]. Hypothesising that these methods could help in setting epidemic thresholds in dengue fever epidemic areas, there were applied in the Central Health Region. Thus, the thresholds set with the mean and C-sum methods were higher than the 2021 monthly incidence rates. It is understandable as the mean (mean + 2 SD), and the cumulative Sum (C-sum + 1.96 SD) are based on the mean incidence rate. They are then subject to excessive monthly incidence rates with higher thresholds. In contrary, the intervention threshold (3rd quartile) was exceeded between July and September by the 2021 incidence rate, based on the median (3rd quartile) method. Quartiles are the best measure for over dispersed data, such as the monthly incidence rates throughout the study period. By the way, July–September 2021 was potentially epidemic, calling for an outbreak investigation to determine the causes. It could go from a mechanical increase due to changes in policies, availability of diagnostic tests or an actual increase in dengue incidence.

This article has shown insightful information about the trends and epidemic thresholds of dengue fever in the Central Health Region—Burkina Faso—between 2016 and 2021. However, the data included clinically suspected cases and probable cases (based on the antigenic and virologic rapid diagnostic tests). It could lead to less accurate thresholds. In addition, there has been a delay in data reporting, due to the reporting system, resulting in difficulties to track epidemics in a real-time basis.

However, this attempt is welcome for policymakers and surveillance professionals in Africa, where dengue fever is likely to be underreported [4]. In addition, a considerable burden of asymptomatic would bring issues about these thresholds based on symptomatic cases. Fortunately, asymptomatic patients seem marginal as a meta-analysis reported 0.0% (95% confidence interval: 0.0–0.5), 3.5% (95% confidence interval: 0.8–7.8), and 15.6% (95% confidence interval: 9.9–22.2) prevalence for ribonucleic acid (RNA), immunoglobulins M and immunoglobulins G [36]. Data were considered monthly in this study. Although epidemics could take weeks, monthly data may prevent the investigator from spotting an epidemic earlier. In a prospective approach, the use of data from the Weekly official letter telegram [Télégramme Lettre Officielle Hebdomadaire: TLOH in French] that reports the cases one week after the other could allow capturing earlier an epidemic.

## Conclusion

The yearly incidence of dengue fever was relatively stable from 2016 to 2021 in the Central Health Region of Burkina Faso. A within-year variability was found during the same period, suggesting an important influence of meteorological factors. The mean and cumulative sum methods were subject to extreme monthly incidence rates and may not be the best approaches for setting dengue fever epidemic thresholds in the region. However, the quartiles method captured an unusual increase in dengue incidence between July and September 2021, calling for outbreak investigations to certify the epidemic and set up necessary responses. In addition, this study is the first to report on the biannual periodicity of dengue fever and set epidemic thresholds in Burkina Faso.

## Data Availability

The data set will be available from the corresponding author upon reasonable request.

## Abbreviations

CDC: Centers for Disease Control and Prevention
CISSE: Centre d’Information Sanitaire et de Surveillance Epidémiologique [Health information and epidemiological surveillance centre]
CM: Centre Médical [Medical centre]
CMA: Centre Médical avec Antenne chirurgicale [Medical centre with surgical antenna]
CSPS: Centre de Santé et de Promotion Sociale [Health and social promotion centre]
C-sum: Cumulative sum
DF: Dengue fever
ENDOS-BF: Entrepôt de données sanitaires du Burkina Faso [Health data depository of Burkina Faso]
IQR (Q1–Q3): Interquartile range (qurtile 1–quartile 3)
SD: Standard deviations
